# Effect of hypothermic perfusion on phacoemulsification in cataract patients complicated with uveitis: a randomised trial

**DOI:** 10.1186/s12886-020-01507-9

**Published:** 2020-06-16

**Authors:** Lu Jiang, Wenjuan Wan, Yan Xun, Liang Xiong, Binge Wu, Yongguo Xiang, Zhouyu Li, Lu Zhu, Yan Ji, Peizeng Yang, Ke Hu

**Affiliations:** 1 Chongqing Medical University, Chongqing, China; 2 The First Affiliated Hospital of Chongqing Medical University, Chongqing Key Laboratory of Ophthalmology and Chongqing Eye Institute, Chongqing, China; 3 The Second affiliated hospital of Baotou medical college, Baotou, Inner Mongolia Autonomous Region China

**Keywords:** Hypothermia, Uveitis, Cataract, Phacoemulsification, Postoperative inflammation

## Abstract

**Background:**

To evaluate the effectiveness and safety of hypothermic perfusion in the phacoemulsification of cataract caused by uveitis.

**Methods:**

This was a prospective, single-masked, randomised, controlled clinical trial. One hundred and six patients with uveitis-associated cataract underwent phacoemulsification with perfusion fluid temperature at 4 °C (treatment group) or 24 °C (control group). Anterior chamber inflammation grade, corneal endothelial cell count, corneal thickness, macular fovea thickness, and intraocular pressure (IOP) were observed on the 1st day and 7th day after operation.

**Results:**

The aqueous flare score was 0.83 ± 0.76 in the 4 °C group, which was lower than that in the 24 °C group (1.51 ± 1.02, *p* = 0.006) on the first day after operation. The aqueous cells score was lower in the 4 °C group (0.17 ± 0.38) than that in the 24 °C group (0.62 ± 0.94, *p* = 0.025). The mean corneal thickness of incision in the 4 °C group (907.66 ± 85.37 μm) was thinner than that in the 24 °C group (963.75 ± 103.81 μm, *p* = 0.005). Corneal endothelial cells density, macular fovea thickness, or percentage of transiently increased IOP showed no difference between the two groups (*p* > 0.05). There was no significant difference in all the main outcome parameters between the two groups on the 7th day after operation (p > 0.05).

**Conclusions:**

Hypothermic perfusion in the phacoemulsification of uveitis-associated cataract is safe, and it can effectively inhibit anterior chamber inflammation and reduce the incisional corneal edema in the early postoperative stage.

**Trial registration:**

The study was registered with the Chinese Clinical Trial Registry. (http://www.chictr.org.cn/, Registration Number: ChiCTR1800016145).

## Background

Uveitis is one of the major causes of blindness worldwide [1]. Certain uveitis patients may complicate with cataract [1, 2]. Cataract in patients with uveitis results from both the process of primary disease and the persistent treatment with corticosteroids [2–4]. Among all the surgical approaches, phacoemulsification is the most effective method, because of a smaller incision of the cornea and a less stimulation of the vitreous [5]. The ultrasound energy applied in phacoemulsification generate the heat, which may cause postoperative anterior chamber inflammation, corneal edema, incision burns, corneal endothelial cells loss, and even decompensation of corneal endothelium [3, 4]. Besides, the postoperative inflammatory reaction of cataracts caused by uveitis is usually more severe than that in senile cataracts because of the adhesive iris or intraocular environment prone to inflammation.

Hypothermia has been proved to be a protective factor to improve the body’s tolerance to ischemia and hypoxia in numbers of studies [6–9]. Since hypothermia could decrease the tissue metabolism to reduce hypoxic-ischemic injury, therapeutic hypothermia is applied in many diseases. In the ocular field, it has been reported in some experiments that local hypothermia can protect the retina from acute ischemic injury and reduce postoperative inflammation in vitrectomy [10, 11]. In cataract surgeries, applying an ice-cold eye mask after operation could increase the comfort level and reduce inflammation with no adverse effects [12]. In our previous study, we found that hypothermic perfusion in the phacoemulsification of senile hard nuclear cataract was safe and could effectively protect the corneal endothelium, decrease corneal edema and reduce anterior chamber inflammation in the early postoperative stage [13]. Therefore, we hypothesized that hypothermia could also protect the cornea during phacoemulsification and reduce the early postoperative anterior chamber inflammation in patients with uveitis.

In this prospective randomised controlled trial, we aim to decrease the temperature of anterior chamber during phacoemulsification to reduce the postoperative reaction in uveitic cataract. Phacoemulsification was performed under an intraocular perfusion temperature at 4 °C (hypothermia) or 24 °C (room temperature) to evaluate the effectiveness and safety of hypothermic perfusion in the phacoemulsification of uveitic cataract surgeries.

## Methods

### Study design

A prospective, randomised, controlled clinical trial was conducted at the department of ophthalmology in the First Affiliated Hospital of Chongqing Medical University, Chongqing, China, a tertiary care center. The trial adhered to the principles of the Declaration of Helsinki. The study was approved by the ethical review committee of the First Affiliated Hospital of Chongqing Medical University, Chongqing, China, and the trial is registered in the Chinese Clinical Trial Registry (registration number ChiCTR1800016145). All the participants provided written informed consent before enrolment. The protocol and the possible risks and benefits of the trial were explained to all the participants.

### Patient selection

The trial started in December 2017 and concluded in May 2019. One hundred and six patients with uveitic cataract were enrolled in this trial. The inclusion criteria were: age of more than 6 years, diagnosis of uveitis-associated cataract, and eyes had no active inflammation for 6 months prior to surgery. The exclusion criteria were: pregnant women, patients enrolled in other clinical studies at the same time, other ocular surgeries at the same time or before, previous usage of contact lenses, patients with other severe ocular diseases, systemic diseases including diabetes or hypertension, and patients who declined to consent.

### Study protocol

The patients were randomly divided into 2 groups (the 4 °C group and the 24 °C group). We chose 4 °C as the treatment group and 24 °C as the control group because 4 °C was available in the refrigerator, and the room temperature in the operating room was 24 °C. The perfusion fluid was stored in the 4 °C refrigerator before the surgery for at least 4 h in the 4 °C group. The perfusion fluid was not pre-treated in the 24 °C group. Both groups underwent phacoemulsification and intraocular lens implantation by the same surgeon. All the eyes were topically anesthetized with benoxinate. Phacoemulsification through a standard clear corneal incision with 2.8 mm in width and 1.5–1.75 mm in length and implantation of an intraocular lens were performed by the same surgeon with the same machine (Stellaris, Bausch & Lomb, USA). The central corneal thickness and the corneal thickness at the incision was measured by anterior segment optical coherence tomography (OCT) (Cirrus HD-OCT 1000, Carl Zeiss Meditec, Inc. Dublin, CA), the macular fovea thickness was observed by fundus OCT (Spectralis OCT, Heidelberg Engineering Corporation, Heidelberg, Germany). Corneal endothelial cell count (Topcon SP-3000P, Topcon Corporation, Tokyo, Japan), slit-lamp examination for the anterior and posterior segment observation, best corrected visual acuity (BCVA) and intraocular pressure (IOP) were examined before operation and on the 1st day and 7th day after operation. BCVA was recorded in Log MAR scores with the standard logarithmic visual acuity chart. Significant clinical visual improvement was defined as an improvement of more than 2 lines on the visual acuity chart [14]. Anterior chamber flare and cells were graded with the Standardization of Uveitis Nomenclature (SUN) Grading System [15] on the 1st day and 7th day postoperatively by a same examiner. Patients and examiners were masked to the grouping. Phacoemulsification time, ultrasound energy and perfusion time were recorded during the surgery. A topical combination of levofloxacin, diclofenac sodium and tobramycin-dexamethasone four times a day and tobramycin-dexamethasone ointment every night was applied after surgery. Systemic medications included prednisone or cyclosporine, and the dose varies according to the patients’ condition.

### Randomization and masking

As a pilot study, we planned to enroll 100 patients instead of calculate sample size. Random numbers were generated by computer. All the patients were randomised to 2 groups before surgery. In order to restrict bias, the study was single-masked. Patients examiners, and evaluating investigators were masked to the grouping. The temperature of perfusate could be sensed by the surgeon during the phacoemulsification, the surgeon was therefore not masked. The surgeon played no other role in the study. To ensure that neither participants nor examiners would know the grouping, the random numbers were kept in envelopes to avoid selection bias. Unmasked patients were withdrawn from this study.

### Statistical analysis

Data analysis was performed using SPSS version 21.0 (SPSS Inc., Chicago, IL, USA). Normality distribution of continuous variables was tested using the Kolmogorov-Smirnov test. The t-test and Mann-Whitney U test were used to compare variables. Pearson Chi-Square test with Fisher’s exact test was applied for analyzing categorical variables. *P*-values lower than 0.05 were considered statistically significant.

## Results

A total of 106 eyes from 106 patients were included in the study. Two patients unmasked were withdrawn from this study. The remaining 106 eyes were randomly divided into 4 °C group and 24 °C group, and each group included 53 eyes. There were no statistical differences in gender, age, systemic anti-inflammatory medicine use, average phacoemulsification time, average ultrasound energy or perfusion time between the two groups (*P* > 0.05) (Table 1).

**Table 1:** Demographic data of study subjects

	4 °C group	24 °C group	T value	*P* value
**Patients**	53	53	N/A	N/A
**Gender**				
**Male**	21 (39.6%)	28 (52.8%)	N/A	0.173
**Female**	32 (60.4%)	25 (47.2%)		
**Age** (year, mean ± SD)	40.2 ± 17.6	42.3 ± 16.9	−0.632	0.851
**Patients using systemic corticosteroids**	45 (84.9%)	43 (81.1%)	N/A	0.605
**Dose of prednisone** (mg/day)	18.5 ± 3.3	18.4 ± 2.8	0.046	0.963
**Patients using cyclosporine**	36 (67.9%)	30 (56.6%)	N/A	0.229
**Dose of cyclosporine** (mg/day)	106.3 ± 16.2	114.2 ± 22.4	−1.613	0.113
**Average phacoemulsification time** (seconds)	20.6 ± 18.0	19.4 ± 21.7	0.239	0.390
**Average ultrasound energy** (%)	5.4 ± 2.3	4.5 ± 2.3	1.554	0.506
**Perfusion time** (seconds)	152.6 ± 62.2	143.3 ± 92.7	0.352	0.548

### Anterior chamber inflammation

**Fig. 1:**
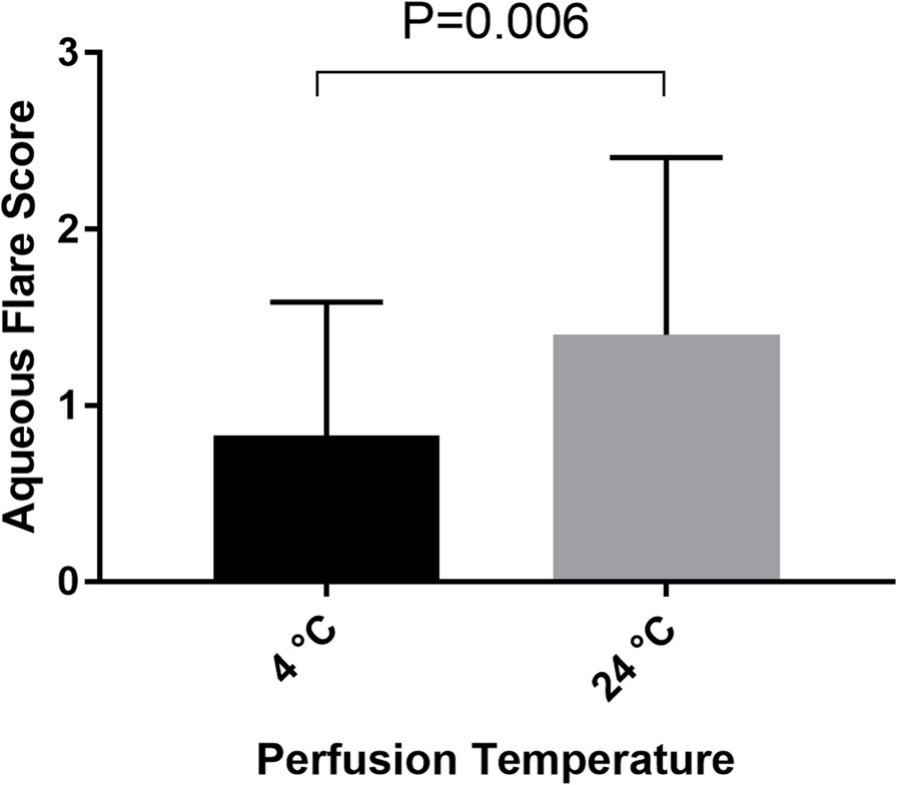
Aqueous flare score measured on the first day after operation in 4 °C group and 24 °C group

**Fig. 2:**
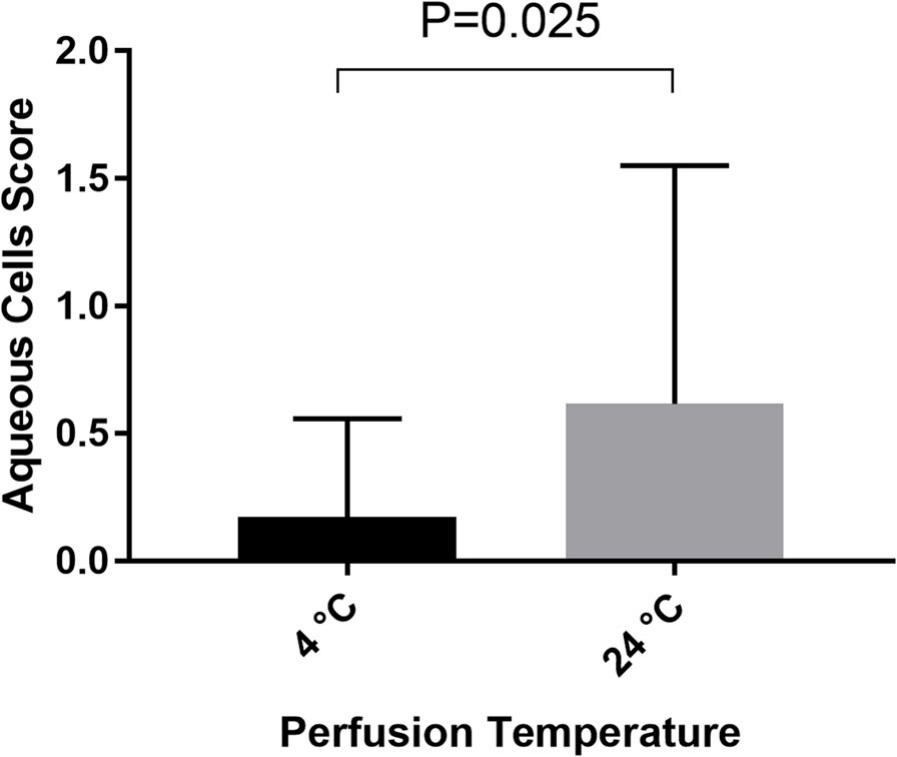
Aqueous cells score measured on the first day after operation in 4 °C group and 24 °C group

The anterior chamber inflammation was described by the aqueous flare and the aqueous cells according to the Standardization of Uveitis Nomenclature (SUN) Grading System. The eyes had no aqueous flare or aqueous cells in the anterior chamber before the surgery. The aqueous flare score was 0.83 ± 0.76 in the 4 °C group, which was significantly lower than that in the 24 °C group 1.51 ± 1.02 (P = 0.006) on the first day after operation (Fig. 1). The aqueous cells score was 0.17 ± 0.38 in the 4 °C group, which was also significantly lower than that in the 24 °C group 0.62 ± 0.94 (*p* = 0.025) (Fig. 2). There was no significant difference in the aqueous flare score or aqueous cells score between the two groups on the 7th day (*p* > 0.05).

### Corneal analysis

The corneal thickness was measured by anterior segment OCT. The mean corneal thickness of incision was 907.66 ± 85.37 μm in the 4 °C group, which was thinner than that in the 24 °C group (963.75 ± 103.81 μm) (*P* = 0.005) on the 1st day post operation. The mean central corneal thickness was 596.53 ± 54.30 μm in the 4 °C group and 603.88 ± 71.12 μm in the 24 °C group, without significant difference between two groups (*P* = 0.654). The corneal endothelial cells were also observed on the 1st day after operation. The mean endothelial cell density was 2845 ± 538.9 cells/mm2 in the 4 °C group and 2473 ± 475.8 cells/mm2 in the 24 °C group (*P* = 0.907). The mean percentage of hexagonal cells was 49.9 ± 12.7% in the 4 °C group and 48.6 ± 12.6% in the 24 °C group (*P* = 0.609). There was no significant difference on the parameters concerning on cornea between the two groups on the 7th day post operation (*p* > 0.05).

### Macular fovea thickness

Because of the cataract and the miotic pupils, some of the macular fovea thickness cannot be observed by OCT before the surgery. The mean macular fovea thickness before surgery was 232.0 ± 26.9 μm in the 4 °C group and 231.6 ± 38.5 μm in the 24 °C group, without significant differences between two groups (*p* = 0.971). On the first day after operation, the mean macular fovea thickness was 232.1 ± 24.3 μm in the 4 °C group and 223.8 ± 34.3 μm in the 24 °C group. There was no significant difference between the two groups (*p* = 0.303). There was no significant difference before and after surgery in the 4 °C group (*p* = 0.989) and the 24 °C group (*p* = 0.480).

### Clinical parameters

Significant clinical visual improvement was defined as an improvement of more than 2 lines on the visual acuity chart. There was no difference between the two groups in the visual improvement on the first day post operation (*p* = 0.183).

Transiently increased IOP was found in 17 eyes in the 4 °C group and 16 eyes in the 24 °C group postoperatively without significant difference (*p* = 0.834). No macular edema, endophthalmitis or other intraoperative or post-operative complications was observed in the two groups during the study.

### Uveitis subtypes

The subtypes of uveitis included Vogt-Koyanagi-Harada (VKH) syndrome (27 patients), anterior uveitis (19 patients), Behcet’s disease (14 patients), pediatric uveitis (11 patients), Fuchs’ syndrome (11 patients), and other subtypes of uveitis (23 patients) which consisted of retinal vasculitis (3 patients), scleritis (1 patient), lymphoma (1 patient), syphilis (1 patient), glaucomatocyclitic crisis (1 patient), panuveitis (1 patient), Blau syndrome (1 patient), and unclassified uveitis (14 patients) (Table 2).

**Table 2:** Subtypes of uveitis

	4 °C group	24 °C group	Total
**Vogt-Koyanagi-Harada disease (VKH)**	11 (20.8%)	16 (30.2%)	27 (25.5%)
**Anterior Uveitis**	13 (24.5%)	6 (11.3)	19 (17.9%)
**Behcet’s disease (BD)**	7 (13.2%)	7 (13.2%)	14 (13.2%)
**Childhood uveitis**	8 (15.1%)	4 (7.5%)	12 (11.3%)
**Fuchs’ syndrome**	5 (9.4%)	6 (11.3%)	11 (10.4%)
**Other subtypes**	9 (17.0%)	14 (26.4%)	23 (21.6%)
**Total**	53	53	106

We found that the mean corneal thickness of incision was 0.874 ± 0.074 mm in the 4 °C group, which was significantly thinner than that in the 24 °C group (0.973 ± 0.116 mm) (*p* = 0.027) on the first day after operation in VKH syndrome patients. Other index including corneal endothelial cells analysis, macular fovea thickness and anterior chamber inflammation were not significantly different (p > 0.05).

## Discussion

Uveitis is a refractory and recurrent disease resulting in blindness. Although phacoemulsification can effectively remove the cataract caused by uveitis, the complexity of uveitis poses for the surgeon special challenges. The cataract caused by uveitis usually complicated with iris atrophy, posterior synechiae, a miotic pupil, pupillary membrane, invisible foveal reflection and bleeding from abnormal fragile iris vessels [16], which leads to additional surgical procedures, develops more intraoperative complications, and has poorer postoperative visual acuity compared with the senile cataract surgery [17]. The postoperative anterior chamber inflammatory reaction is usually more serious than senile cataract [18]. We therefore aim to find a method to alleviate the inflammatory response after phacoemulsification in cataract eyes complicated with uveitis. In our study, hypothermic perfusion was used to decrease the anterior chamber temperature during phacoemulsification to reduce postoperative inflammatory response. The results showed that compared to 24 °C (room temperature), perfusion fluid at 4 °C during phacoemulsification was safe, and it resulted in milder anterior chamber inflammatory reaction and incisional corneal edema in the early postoperative stage.

Hypothermic perfusion could alleviate postoperative inflammatory responses. In our study, the hypothermic perfusion group had milder anterior chamber inflammatory reaction in the early stage after cataract surgery than the room temperature group. This might be due to the hypothermic perfusion in the anterior chamber that increased the tolerance of the anterior segment to ischemia and hypoxia, or decreased cellular metabolism. Hypothermia has been applied in many fields. It has been shown that transient hypothermia is an effective factor to induce tolerance to ischemic or hypoxic injury in nervous and cardiac systems [19, 20]. Local hypothermia has been also proved to protect the retina from acute ischemic injury caused by increased IOP and reduce postoperative inflammation in vitrectomy [10, 11]. Hypothermia could protect retinal ganglion cells against an ischemic insult and prolongs their ischemic tolerance time [21]. It has been demonstrated that the neuronal energy metabolism has been reduced by hypothermic preconditioning [22]. Meanwhile, hypothermia could decrease cellular metabolism, reduce vascular endothelial growth factor-A (VEGF-A) and sustain pigment epithelium–derived factor (PEDF) expression to create an anti-angiogenic environment in culture of human retinal pigment epithelium (RPE) cells [23]. The global or ocular hypothermic preconditioning could also significantly protect retina from ischemia/reperfusion injury by reducing retinal glutamate uptake and glutamine synthetase activity [24]. Cold exposure induces the expression of cold-shock proteins to inhibit the apoptotic process and make the visual neurons survive the retrograde insult [25].

In addition, it has been observed that hypothermia could alleviate postoperative corneal edema at the incision, especially in eyes with VKH syndrome. This might be because hypothermic perfusion can reduce thermal damage caused by phacoemulsification. Ultrasonic energy in phacoemulsification is used to emulsify the lens nucleus. The vibration of the metal tip creates friction and generates heat. Excessive heat burns the cornea, causing corneal edema, melting and scarring of cornea [26]. In our experiments, the temperature of the perfusate which took away some of the heat generated by phacoemulsification has been decreased to reduce incisional corneal edema at the early postoperative stage.

In our previous work, similar study of hypothermic perfusion in hard nuclear senile cataract by animal experiments and clinical trials has also been performed [13]. There was a significant difference in the central and incisional corneal thickness, the corneal endothelial cells loss, and the anterior segmental inflammation between the 4 °C group and the 24 °C group in hard nuclear cataract surgeries. However, compared with reducing corneal edema, hypothermia tended to play a more vital role on alleviating anterior chamber inflammation in uveitic cataract. There were some reasons to explain the results. Firstly, the cataract caused by uveitis generally occurred in younger patients in comparison with senile cataracts [27]. The most common subtype would be posterior sub-capsular cataracts instead of nuclear sclerosis cataracts [28]. Thus, it required shorter phacoemulsification duration and less ultrasound energy, leading to milder corneal damage. Secondly, postoperative inflammatory reaction was usually more serous in uveitis complicated cataract as compared with senile cataract. Thirdly, the timing of the surgeries was strictly controlled to avoid severe intraocular inflammation occurred post cataract operation. It was generally recommended that a successful cataract surgery complicated with uveitis requires a quiet eye devoid of active inflammation for at least 3 months [29]. In our study, all the patients were followed-up for at least 6 months before the surgery to ensure there was no active inflammation preoperatively. That was the key point to reduce postoperative inflammatory reaction. Fourthly, perioperative medication was also important. The patients were given adequate perioperative prophylactic anti-inflammatory therapy by a topical combination of diclofenac sodium and tobramycin-dexamethasone, as well as systemic prednisone and/or cyclosporine. It was recommended to use supplemental corticosteroids and immunosuppressive agents to control postoperative inflammation and reduce the recurrence of uveitis [27–29].

The results of our trial showed that hypothermic perfusion was effective in reducing the early inflammatory response after cataract surgery of eyes with VKH syndrome. Uveitis associated with VKH syndrome is an autoimmune disease which progresses to chronic recurrent granulomatous intraocular inflammation. Patients with VKH syndrome are more likely to have ocular complications, such as cataract, high intraocular pressure, and fundus lesions, which lead to poor visual prognosis [30, 31]. It was reported that the incidence of cataract in patients with VKH syndrome ranges from 5 to 45% [32]. Chronic recurrent VKH syndrome had a worse cataract surgery prognosis. We therefore need to reduce the inflammatory response after cataract surgery to minimize recurrences of uveitis. Therefore, we recommend to use the hypothermic perfusion fluid to reduce anterior chamber inflammation and incisional corneal damage at the early postoperative stage for VKH syndrome cataract patients.

There were several limitations in our study. The trial was single-masked (participants and examiners were masked while the surgeon was not), which could not completely avoid the subjective bias from the researchers. Besides, the anterior chamber inflammation was measured using the SUN Grading System, which might increase the bias of subjective interpretation. Laser flare and cell meter would be more objective and will be conducted in the next step. In this study, the clinical parameters have been observed in the early postoperative stage. The follow-up period should be extended to observe long term postoperative reactions including macular changes in future studies. Further study with accurate record of perfusion amount and its effect on endothelial cell loss would be conducted. And further multi-center large sample study by different tertiary centers would be necessary to analyze the beneficial effects of intraoperative hypothermic perfusion on different subtypes of uveitis.

## Conclusions

In conclusion, hypothermic intraocular perfusion during the phacoemulsification is safe, and it can effectively inhibit the anterior chamber inflammation and reduce corneal edema at the incision at the early postoperative stage in cataract patients with uveitis, which could be recommended in the uveitic cataract surgeries.

## Data Availability

The datasets analysed are available from the corresponding author on reasonable request.

## Abbreviations

IOP: Intraocular pressure
OCT: Optical coherence tomography
BCVA: Best corrected visual acuity
SUN: Standardization of Uveitis Nomenclature
VKH syndrome: Vogt-Koyanagi-Harada syndrome
VEGF-A: Vascular endothelial growth factor-A
PEDF: Pigment epithelium–derived factor
RPE: Retinal pigment epithelium.
